# Supplementary material for: NETosis and Neutrophil Activity Quantification in Pediatric Patients with Essential Thrombocythemia
Source: Int J Mol Sci. 2025 Dec 11;26(24):11958. doi: 10.3390/ijms262411958 (PMC12732377; doi:10.3390/ijms262411958)
Supplement: Supplementary file 1 [file ijms-26-11958-s001.zip › ijms-3987961-supplementary.pdf]

Supporting Information

NETosis and neutrophil activity quantification in pediatric patients with essential thrombocythemia

Ekaterina-Iva A. Adamanskaya[1,2], Julia-Jessica D. Korobkin [2], Alexey V. Pshonkin[1], Alexey V Bogdanov[1], Sofia V. Galkina[1,2], Nadezhda A Podoplelova[1,2], Eugenia V. Yushkova[1,2], Mikhail A. Panteleev [1,2,3], Galina A. Novichkova[1], Nataliya S Smetanina[1], Anastasia N. Sveshnikova[1,2,3,*]

[1] Dmitry Rogachev National Medical Research Center of Pediatric Hematology, Oncology and Immunology, Moscow, Russia

[2] Center for Theoretical Problems of Physico-Chemical Pharmacology, Russian Academy of Sciences, Moscow, Russia

[3] Lomonosov Moscow State University, Moscow, Russia

***Corresponding author:** Anastasia N Sveshnikova, a.sveshnikova@physics.msu.ru

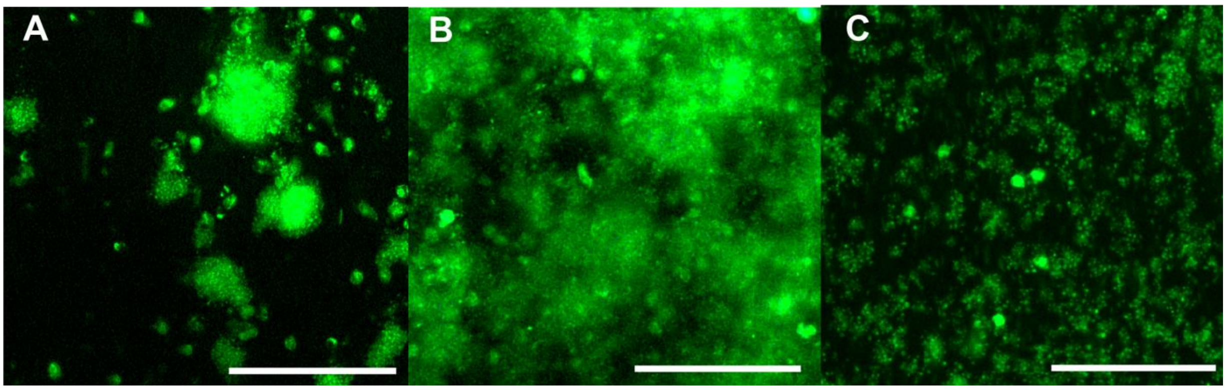

Figure S1. Typical thrombi micrographs for healthy donor (A), typical non-diluted sample from ET patient (B), and typical diluted sample from ET patient (C) at 25 minutes. Scale bar 100 µm. Green – DiOC6

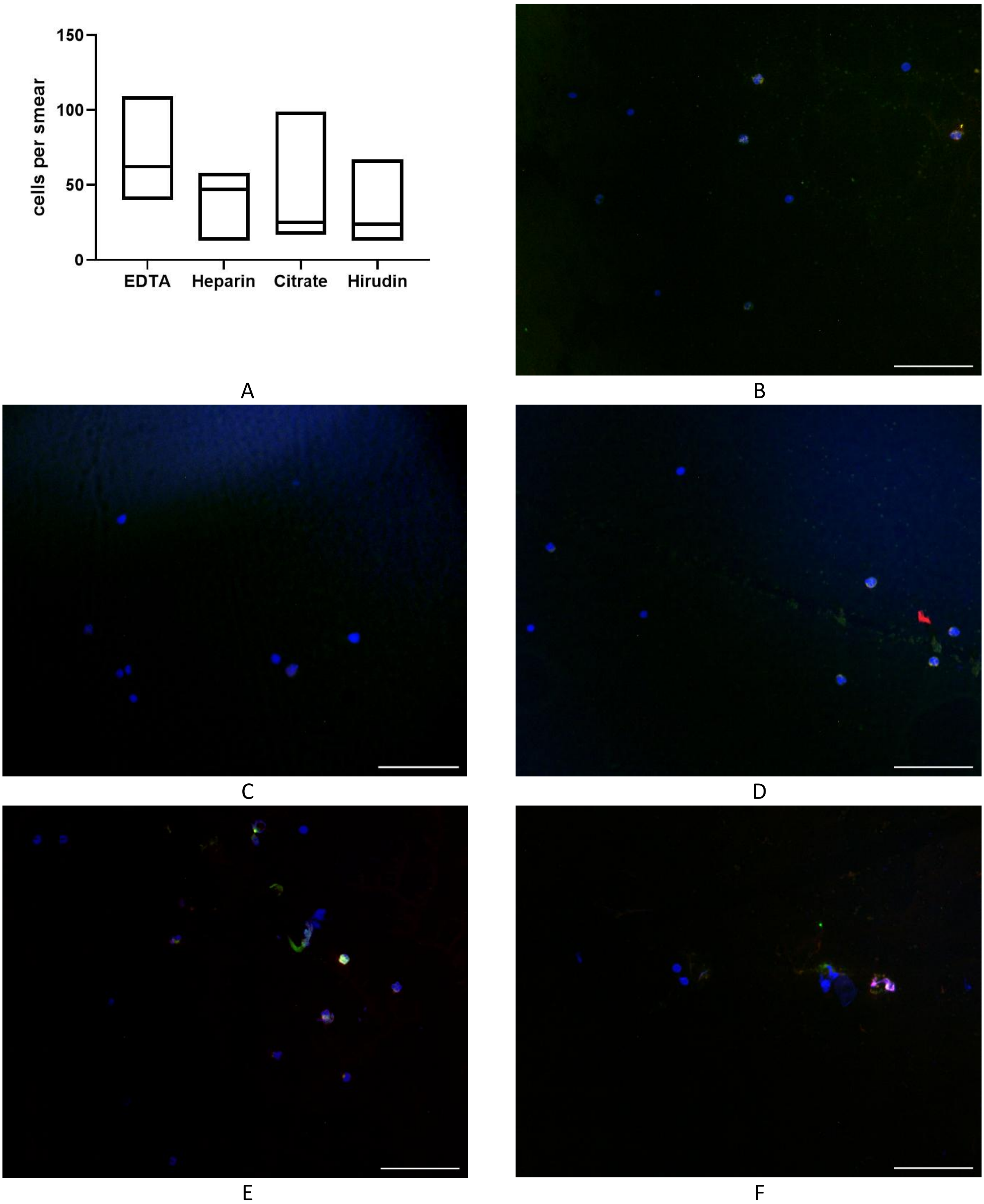

Figure S2. *Anticoagulation impact on cell number in blood plasma smears.* A. Comparison between different anticoagulants (*n* = 3). (B-D) Typical fields of view of smears made from healthy donors blood samples anticoagulated with EDTA (B), sodium citrate (C), or heparin (D). (E-F) Typical cell nuclei deformation observed in heparin (E) and hirudin(F) anticoagulation. Blue – DNA (Hoechst 33342), green - MPO, red is ELA (neutrophil elastase), scale bar 100 µm.

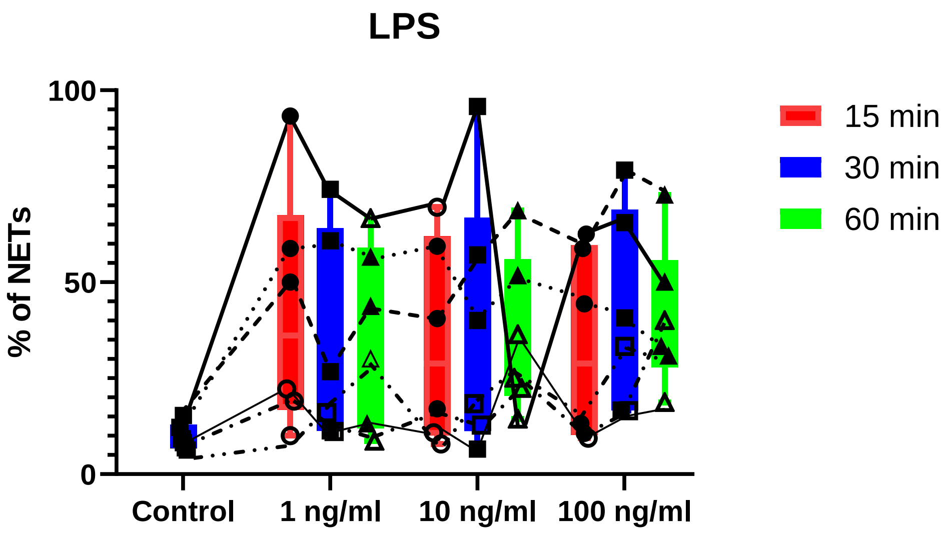

A

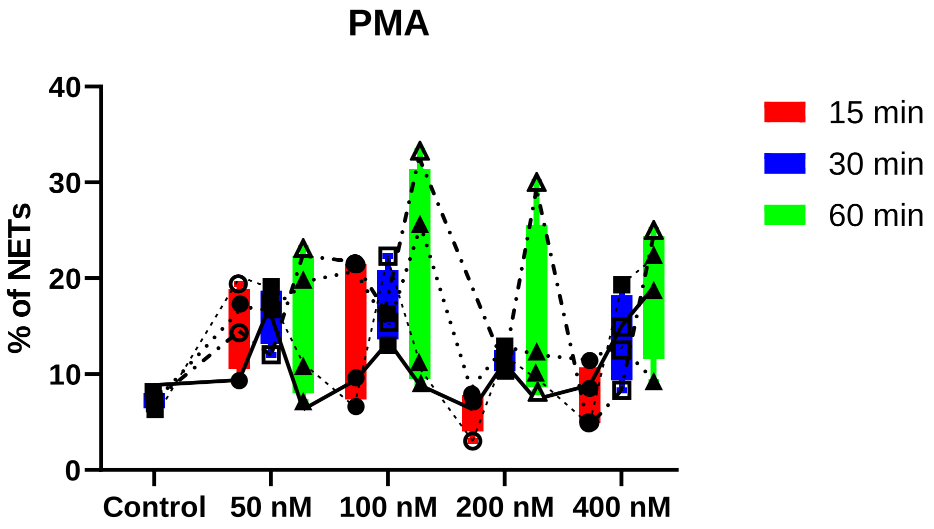

B

Figure S3. *Level of NETosis in healthy donors plasma smears from samples stimulated with different concentrations of LPS (A) and PMA (B) for 15-60 min*. A. Level of NETosis in response to LPS. B. Level of NETosis in response to PMA. Filled symbols indicate experiments where total granulocyte count was more than 50 cells per 100 FOVs. The lines indicate data for individual donors.

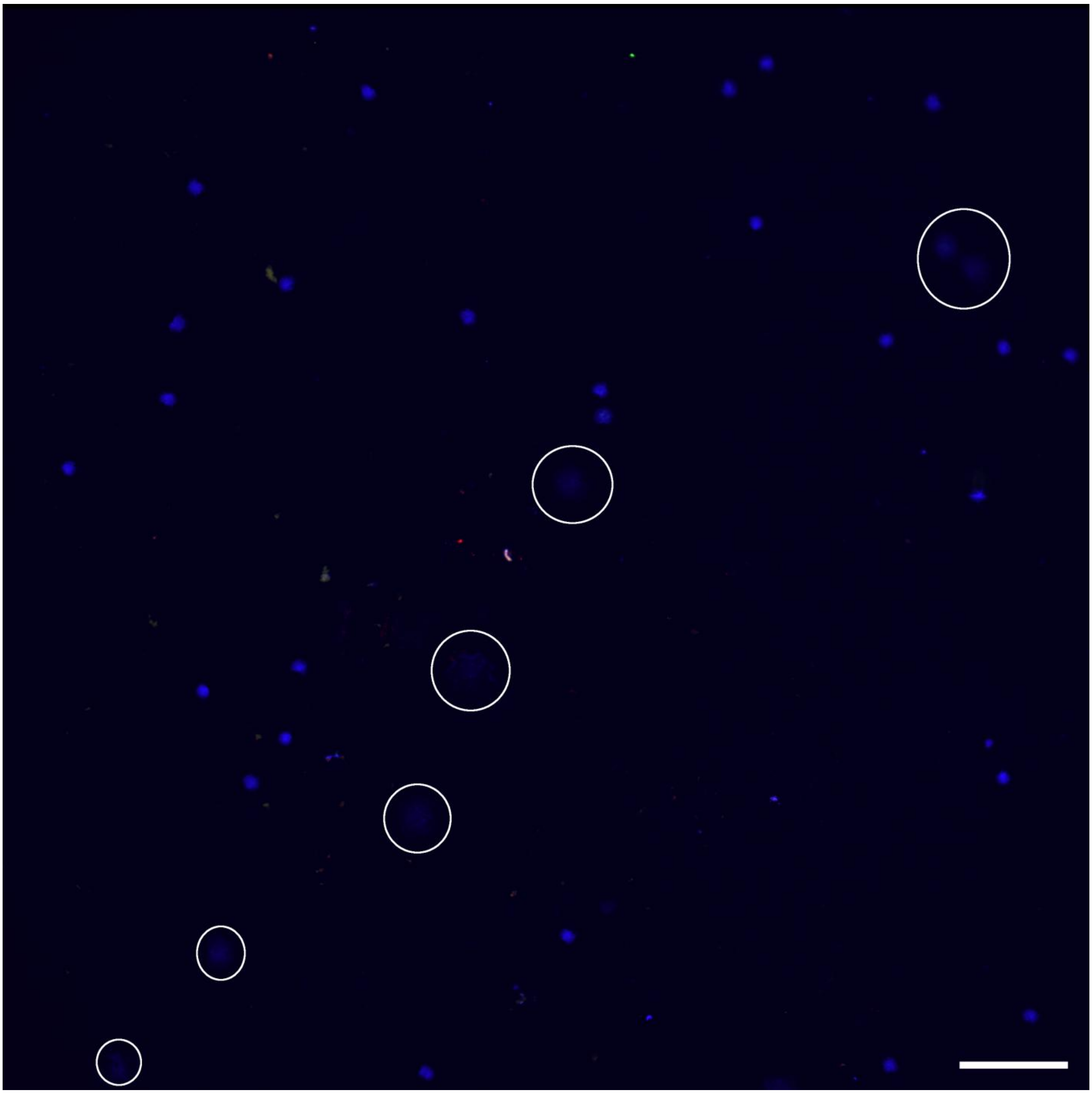

Figure S4. Typical FOV of patient J7. Most net-like structures do not contain MPO or ELA. Blue – DNA (Hoechst 33342), green - MPO, red is ELA (neutrophil elastase), scale bar 100 µm.

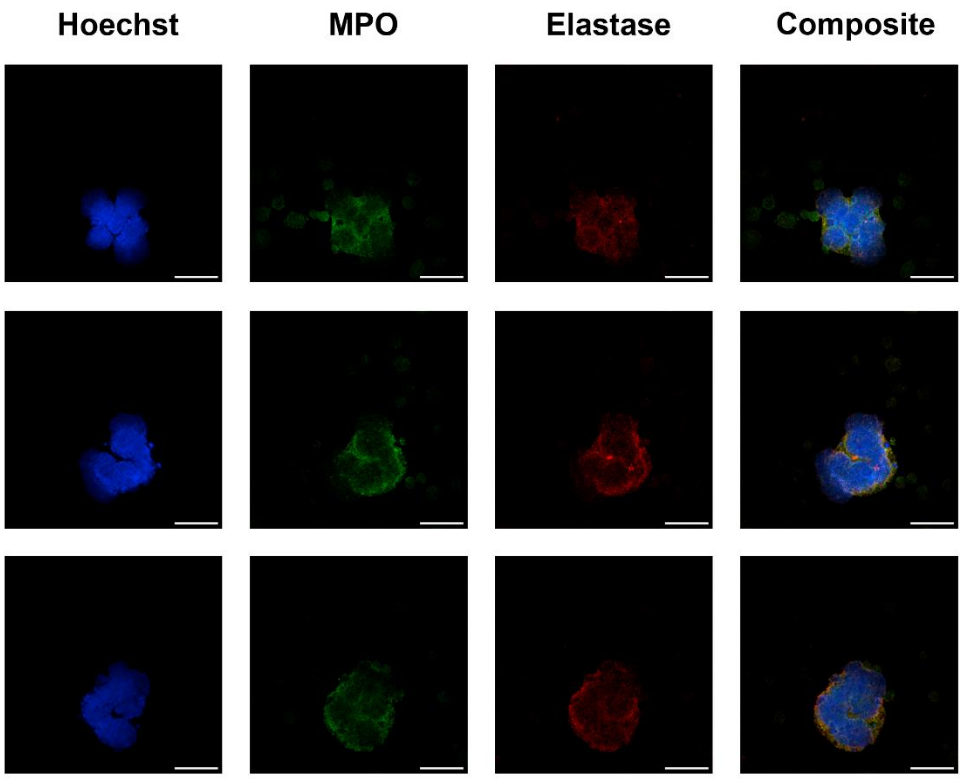

C6

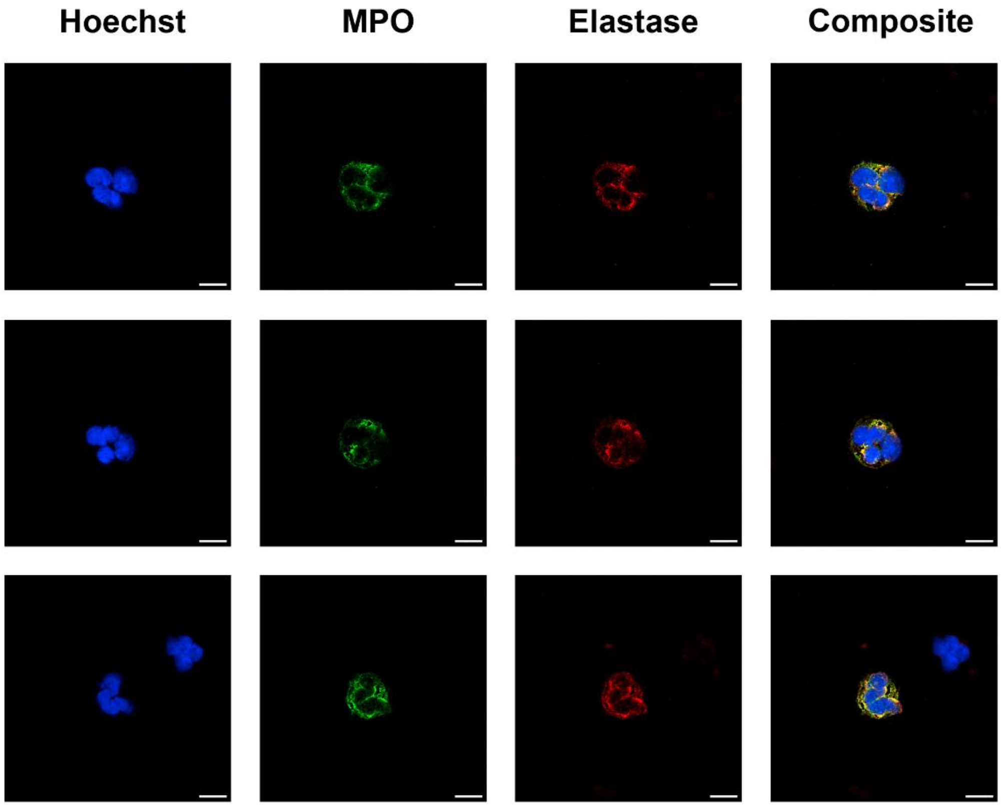

J7

Figure S5. DNA halo around cells in some patients. Blue – DNA (Hoechst 33342), green - MPO, red is ELA (neutrophil elastase), scale bar 10 μm.

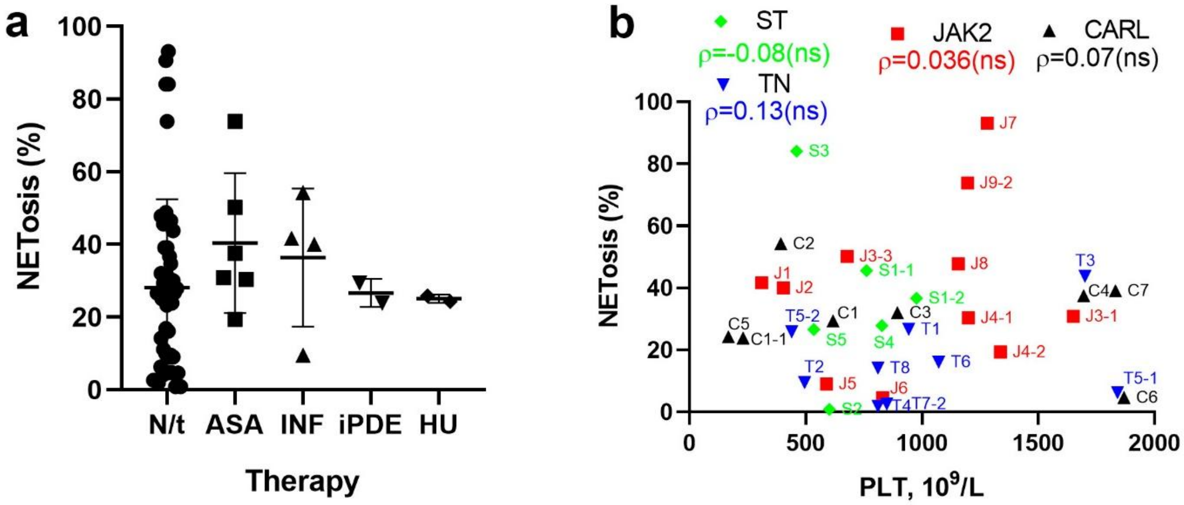

Figure S6. a: Association between NETosis and the applied therapy: anagrelide (iPDE), acetylsalicylic acid (ASA), pegylated interferon alfa-2a (INF), hydroxyurea (HU). b: Association between NETosis and platelet count (PLT), Spearman correlation coefficient (ρ) and its significance are given. ns – non significant ($p > 0.05$).

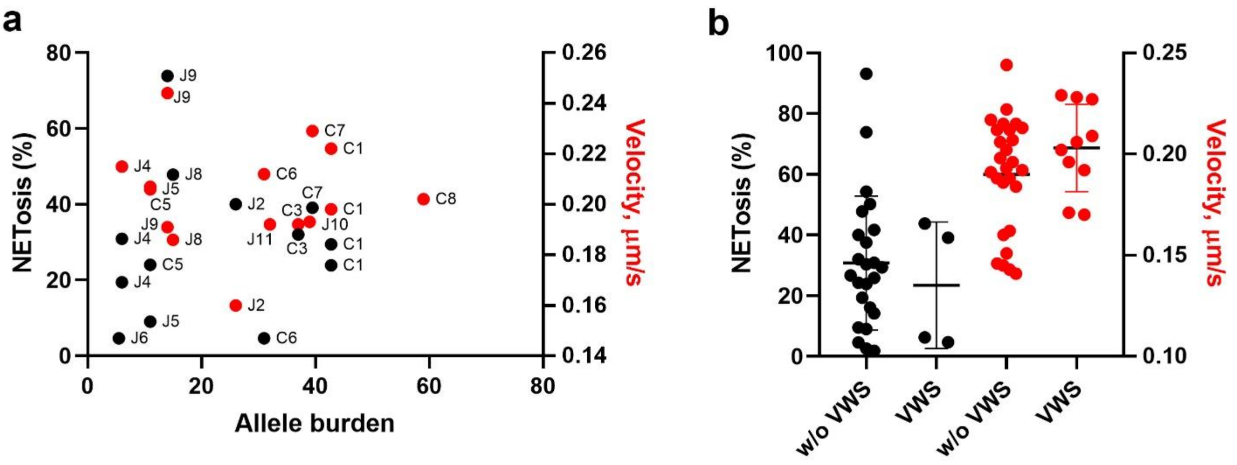

Figure S7. a: Allele burden impact on NETosis and average neutrophil movement velocities. Dots labeled with the corresponding patient ID (Table 1). b: Association between NETosis and average neutrophil movement velocities and acquired von Willebrand syndrome.
